# HPnet: Hybrid Parallel Network for Human Pose Estimation

**DOI:** 10.3390/s23094425

**Published:** 2023-04-30

**Authors:** Haoran Li, Hongxun Yao, Yuxin Hou

**Affiliations:** School of Computer Science and Technology, Harbin Institute of Technology, Harbin 150001, China; haoran_li@hit.edu.cn (H.L.); yuxinhou_054@outlook.com (Y.H.)

**Keywords:** human pose estimation, hybrid parallel model, cross-branches attention, complementary capability, semantic conflict

## Abstract

Hybrid models which combine the convolution and transformer model achieve impressive performance on human pose estimation. However, the existing hybrid models on human pose estimation, which typically stack self-attention modules after convolution, are prone to mutual conflict. The mutual conflict enforces one type of module to dominate over these hybrid sequential models. Consequently, the performance of higher-precision keypoints localization is not consistent with overall performance. To alleviate this mutual conflict, we developed a hybrid parallel network by parallelizing the self-attention modules and the convolution modules, which conduce to leverage the complementary capabilities effectively. The parallel network ensures that the self-attention branch tends to model the long-range dependency to enhance the semantic representation, whereas the local sensitivity of the convolution branch contributes to high-precision localization simultaneously. To further mitigate the conflict, we proposed a cross-branches attention module to gate the features generated by both branches along the channel dimension. The hybrid parallel network achieves 75.6% and 75.4% *AP* on COCO validation and test-dev sets and achieves consistent performance on both higher-precision localization and overall performance. The experiments show that our hybrid parallel network is on par with the state-of-the-art human pose estimation models.

## 1. Introduction

Human pose estimation methods devoted to localizing the pre-defined anatomical keypoints of the person in the still images [1,2], which is a fundamental task in the field of computer vision. It is widely applied to action recognition [3], action forecast [4], human-computer interaction [5,6], etc. Over the past years, the deep convolutional neural networks [1] have achieved impressive performance in the field of human pose estimation and become the mainstream models. Although the prevailing deep convolutional neural networks pre-trained on large ImageNet datasets are adapted as backbones for human pose estimation [7,8,9], these methods still suffer from the large variance of human pose. Due to the spatial locality of the convolutional neural network, increasing the depth of convolutional models solely is inefficient to mitigate the effect of large variances. Modeling the internal dependency of data explicitly contributes to alleviating the large variances. Moreover, the human pose is a well-defined structure and possesses strong mutual dependency on each keypoint.

To model the dependency of human pose, the transformer models [10,11], which consist of self-attention modules, are customized to explicitly model the long-range dependencies over the entire image space. The existing hybrid models [12,13] injected the self-attention module into a pure convolutional neural network to model the long-range dependency. These models conventionally stacked the self-attention modules after the convolutional neural networks, and facilitate estimating human poses even with large variances. The TransPose [12] proposed a sequential model, which stacked the self-attention blocks at the tail of the CNN-based model. The PET [13] proposed a fast sequential model with the same strategy. Although the hybrid models have achieved considerable improvement in human pose estimation, the high-precision localization is still unsatisfactory. The TransPose [12] achieved decent 1.5% improvement on AP, but only gained 0.5% on AP75. The reason is that hybrid sequential models leverage the dependency modeling ability of the self-attention model but lack taking advantage of the property of convolution.

According to our observation, the convolutional module is sensitive to the local pattern but ineffective in modeling long-range dependency, whereas the self-attention module tends to model long-range dependency but is detrimental to high-precision localization. The existing hybrid sequential models tend to cause mutual conflict, which amplifies the property of self-attention to dominance and suppresses convolution. Thus, the long-range dependency modeling capability is boosted by the self-attention module; nevertheless, the local sensitivity is degraded. As a result, the average precision is improved, but the AP75 is still unsatisfied. Exploring a new structure of hybrid models is a key role to mitigate this mutual conflict and leverage both complementary capabilities.

To mitigate this mutual conflict, this paper presents a new hybrid model named Hybrid Parallel network (HPnet) to leverage the complementary capability simultaneously. In contrast to the previous hybrid sequential models, our model processes the features separately throughout the pipeline, which encourages each branch to learn internal property without interruption. Furthermore, we develop a cross-branches attention block to fusion the two types of features, while it is contributing to mitigating the semantic conflict between these two types of features. Compared to previous hybrid sequential models, our model achieves consistent improvements on overall AP and AP at a high *OKS* threshold.

The contributions are summarized as follows:

- We propose a novel Hybrid Parallel network (HPnet) to localize the keypoints. The HPnet leverages the capabilities of the self-attention-based model and CNN-based model.
- We develop a cross-branches attention block(CBA) to fusion the parallel features generated by both branches. The cross-branches attention mitigates the semantic conflict.
- We evaluate our model on the COCO keypoints dataset, and the performance is comparable to the state-of-the-art methods.

## 2. Related Works

### 2.1. Human Pose Estimation

The convolution-neural-networks-based human pose estimation methods [1,14,15] achieved remarkable performance. The Deeppose [14] model first adopted a fully convolutional neural network to directly regress the locations of the human poses. To achieve high-precision human pose estimation, the CPM [1] predicted the confidence heatmap of human keypoints rather than the coordinates, which became the prevailing architecture in HPE. The following methods [7,8,16] transferred the backbone model pre-trained on the ImageNet and then designed a specific architecture to generate precision heatmaps of each joint. Hourglass [16] designed a multi-stage hourglass-like network to refine the confidence heatmap of the human pose. CPN [7] utilized multi-scale features and developed a refiner to produce high-precision heatmaps. HRNet [9] designed a new deep high-resolution convolutional network for heatmap-based pose estimation, which utilize the multi-scale features in each stage to maintain the resolution of features. The SimpleBaseline [8] proposed a simple but efficient encoder–decoder network to facilitate localizing keypoints. Recent research [17] revisited regression-based human pose estimation methods and proposed a more powerful flow-based loss to facilitate the keypoints regression. All the CNN-based models gradually enlarge the receptive field to model long-range relationships implicitly and tend to model the local compact relations.

### 2.2. Hybrid Models

In recent years, the transformer [10,11]-based models which consist of stacked self-attention blocks burst in the field of the natural language process and computer vision. Differing from the CNN, the transformer models the arbitrary range of dependencies in the source space for each layer within the model, which extends the model capacity even in the shallow model. In the NLP, the BERT [18] and GPT-2 [19] are devoted to training a large transformer in a self-supervised way, which maximizes the model capacity. In the computer vision tasks, the VIT [20] proposed the vision transformer for image classification and transfer to pre-text vision task, and the PIT [21] is devoted to building a common architecture for low-level image process. For human pose estimation, the hybrid models [12,22,23,24,25,26] adapted a transformer into a convolution neural network architecture to boost the semantic representation. SwinPose [25] adopted the swin-transformer [27] for pose estimation. ToKenPose [26] employed a standard VIT architecture to detect keypoints. These pure transformer-based models take the advantage of the large model capacity without task prior knowledge, which tends to utilize huge models to localize the keypoints accurately. The hybrid model, which combines self-attention and convolution, defines a compromise solution for human pose estimation, while it leverages the model abilities of both types of models. The TransPose [12] proposed a sequential model, which stacks the self-attention blocks at the tail of the CNN-based model. The PET [13] proposed a fast sequential model with the same strategy. The Poseur [28,29] adopted a ViT model to directly regress the human pose. However, the hybrid sequential models do not leverage the ability of both types of models effectively.

### 2.3. Attention Mechanism

The attention module [30] aimed to model the significance of features and mainly focused on the channel dimension and spatial dimension. The SEnet [31] developed channel-wise attention to enhance the representative features. The Fcanet [32] extended the channel attention into multiple frequency domains. The CBAM [33] adopted spatial attention to filter unimportant regions. The PSANet [34] proposed bi-direction spatial attention to relax the local neighbor constraint. The STAT [35] adopted spatial-temporal attention to further catch the significant regions in the video. For multi-branches models [36,37,38,39,40], these methods facilitated the feature aggregation with simple addition. The PATN [4] proposed element-wise attention to fuse features generated by the dual path network. The mechanism of feature aggregation of a different branch of the network is still unclear, and the previous works generally utilized the trivial addition to fusion.

## 3. Method

### 3.1. Overall Framework

The goal of this paper is to present a paradigm to construct a hybrid parallel network, which typically involves self-attention blocks and variant CNN-based blocks. Differing from the hybrid sequential models such as Transpose [12] which stack the self-attention blocks after the CNN-based blocks, we adopted a parallel strategy to construct the model. Inspired by ResNet [41], the hybrid parallel network consists of three parallel blocks, and each block inherits the same structure.

**Figure 1:**
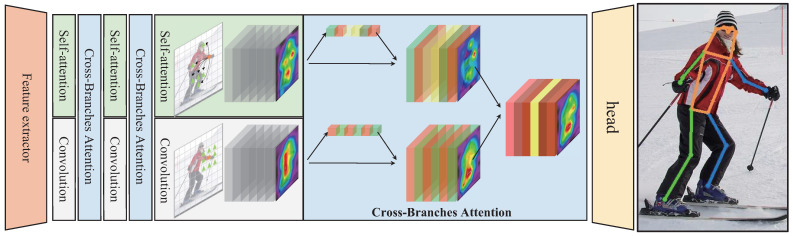
The framework of our HPnet. The HPnet consists of a CNN-based feature extractor and multiple parallel blocks. Each parallel block consists of three elements: the self-attention branch, the convolution branch, and the fusion block.

As illustrated in Figure 1, we proposed a Hybrid Parallel network (HPnet), which consists of a CNN-based shallow feature extractor and multiple parallel blocks. Each parallel block consists of three elements: the self-attention branch which models the arbitrary range dependency, the convolution branch which models the local dependency gradually, and the fusion block which aggregates different ranges of dependencies. Compared to the SimpleBaseline [8], we adopt the self-attention branch as a parallel branch and a fusion module to aggregate both features. The convolution branch is following the conventional ResNet [41], and the head for pose heatmaps is the same as the SimpleBaseline [8]. Differing from the transPose [12], we enforce the self-attention branch to learn complementary features rather than modeling one type of dependency in the sequential model.

For convenience, we use $X_{t}$ to represent the features generated by the self-attention branch, $X_{r}$ to represent the features generated by the convolution branch and $X_{f}$ to represent the aggregated features.

### 3.2. The Parallel Branches

The self-attention branch and the convolutional branch are separated and transform the features independently. To learn multi-scale features, the down-sampling module in each module is adapted to downscale the features, which is a bottle-neck residual block with stride=2.

**Figure 2:**
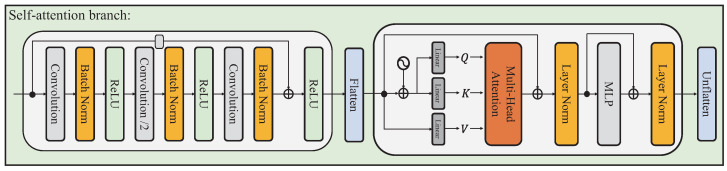
The detail of the self-attention branch.

This self-attention branch consists of a down-sampling module and multiple self-attention modules, which are adopted from the standard transformer encoder [10]. Due to the high computational cost of the self-attention module, the down-sampling block is adopted to downscale the features to alleviate this cost. Moreover, this block also aligns the channel dimensions of the features with the convolutional branches. As shown in Figure 2, the down-sampling module is a pre-activate residual block, which consists of three convolution operators and the stride of the second convolution operator is set to 2. To model the long-range dependency, the following self-attention module, which consists of a multi-head self-attention module and a feedforward network, is adopted to calculate the global similarity and incorporate the semantic information of the entire spatial dimension of features. In addition, the self-attention module computes the dependency of each feature, which also reveals the structure relation of the human pose in this task.

Given an input 2D spatial feature $X_{f}\in R^{C\times H\times W}$, the down-sampling module generates a new feature $X_{t}\in R^{c\times h\times w}$, and h=H/r,w=W/r. Here, we set r=2 for each parallel block. As the input of the self-attention block is a 1D spatial feature, we flatten the feature into the 1D form $X_{t}\in R^{c\times hw}$. The self-attention module first generates the three features as query *Q*, key *K*, and value *V*, and then feeds into a multi-head self-attention block formulated as:

(1) $$\begin{array}{cc} X_{t}^{\prime } & =X_{t}+\mathrm{MHSA}\left(Q,K,V\right) \end{array}$$

(2) $$\begin{array}{cc} X_{t} & =X_{t}^{\prime }+\mathrm{FFN}\left(\mathrm{LN}\left(X_{t}^{\prime }\right)\right) \end{array}$$

The MHSA is a multi-head self-attention module, LN is a layerNorm operator, and the FFN is two layers perception. The final unflatten operator transforms the feature into $X_{t}\in R^{c\times h\times w}$. The MHSA mainly models the global similarity, which concatenates several self-attention of the different subspace of features. The self-attention operator is formulated as:

(3) $$X_{t}^{\prime }=\mathrm{softmax}\left(\frac{QK^{T}}{\sqrt{d_{k}}}\right)V$$

The self-attention branch generated the features, which possess long-range semantic information. This information promotes the semantic representation of each feature and improves the recall of the human pose. However, the process of generating global similarity in the self-attention module discards the spatial relations, resulting in local structural insensitiveness. Therefore, a convolutional branch is utilized to mitigate the insensitiveness.

### 3.3. The Convolutional Branch

**Figure 3:**
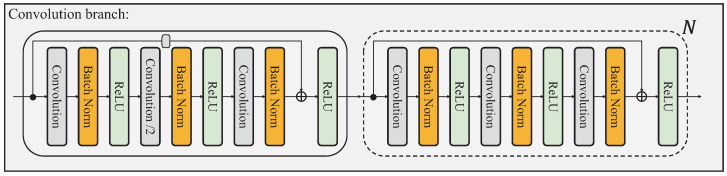
The detail of the convolutional branch.

To generate locally sensitive features, we modify a ResNet-like convolutional block and plugin multiple blocks into each convolutional branch. As illustrated in Figure 1, we divide the classic ResNet [41] into a plain convolutional stage and four residual convolutional stages. In this paper, the last 3 stages are modified into HPnet and the remaining stages are treated as the feature extractor. Thus, the convolutional branch consists of a down-sampling ResBlock with stride=2 and a fixed number of ResBlock with stride=1 illustrated in Figure 3. Given the input feature $X_{f}\in R^{C\times H\times W}$, the module encodes the feature into $X_{r}\in R^{c\times h\times w}$. The number of blocks in each stage is following the original ResNet.

### 3.4. The Cross-Branches Attention

The parallel branches generate two types of features, while the features imply distinctive inherent modalities of dependency. The convolutional branch progressively enlarges the receptive field, meanwhile, it is sensitive to the location. As a result of these properties, the feature $X_{r}$ generated by the convolutional branch captures the local structure, which facilitates estimating human pose accurately. On the contrary, the self-attention branch establishes the global dependency by enumerating the entire spatial location of feature space, thus it is insensitive to the local structure. Even the positional encoding is injected to overcome this weakness, the self-attention branch is still unsatisfied with the high-precision location. Therefore, fusing these two types of features leverages complementary properties.

Although the parallel strategy avoids the dominance of one type of feature compared to the sequential model, the mutual conflict still remains by simply adding these features. The addition treats these features as consistent features, which disrupts the internal properties of these two features. To mitigate the conflict, we design a cross-branches attention module, which constructs two soft-gated functions to monitor the features. In general, the cross-branches attention module is formulated as:

(4) $$X_{f}=G_{t}⊙X_{t}+G_{r}⊙X_{r}$$

The key role of this module is to determine the way to generate the gated value G. To define this attention module [34,35,42], we first explore the dimension of the gate and then investigate the features to generate the gate value. The existing spatial attention [33] generates a gated value for each location, which implies the different importance of each location for the task. By contrast, the channel attention [31] produces a gated value for each channel, which means the different channels of the features tend to represent one specific semantic information, and each type of semantic information is supported by a variant range of dependencies. Thus, the channel-wise gated function is adopted to fusion different ranges of dependencies.

In the existing dual path methods [4], the feature to generate the gate value for one path is from another path, which generally fuses the multi-modality data. Differing from these methods, our model aggregates the features generated from one modality with different properties. This way induces a mutual fusion rather than a complementary fusion. To establish a complementary fusion module, we develop a cross-branches module.

**Figure 4:**
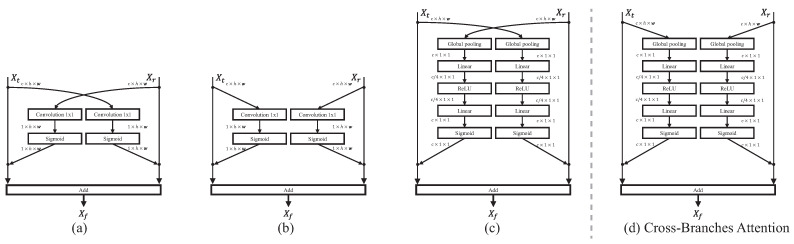
The detail of different attention modules. (**a**) is mutual spatial-based attention, (**b**) is spatial-base attention, and (**c**) is mutual channel-based attention. The (**d**) is our cross-branches attention module.

As shown in Figure 4, the gated module consists of five basic operators. The features are down-sampled to $X_{g}\in R^{C\times 1\times 1}$ with a global average pooling operator, and then with a two layers perception to boost the feature representation.

(5) $$G=\mathrm{sigmoid}(\mathrm{MLP}\left(X_{g}\right))$$

Finally, we adopt the sigmoid function to map the value of features to (0, 1). Both features are gated by the corresponding gated value and then added together. The cross-branches attention module generates aggregated features, that are fed into the next stage.

In the three stages of parallel modules, the resolution of features is downscaled to 1/32 of the original input image. Following the SimpleBaseline, we utilize a three-layer transpose convolutional operator as the output head to upsample the feature heatmaps to 1/4, which facilitates the high resolution of the output confidence map.

### 3.5. Loss

The hybrid parallel net generates the heatmap of the human pose, and we adopt a joint MSE loss to end-to-end train this model. Given the target heatmap H, our model generates the heatmap $\hat{H}$ to predict. The loss is formulated as:

(6) $$L=\frac{1}{KHW}\sum_{khw}I_{k}{\left({\hat{H}}_{khw}-H_{khw}\right)}^{2}$$

Here, the *K* is the number of joints, and $I_{k}$ means the visibility of each joint. This conventional heatmap loss leads to a competitive performance without any hyper-parameters.

## 4. Experiments

We conduct experiments on the COCO [43] person keypoints dataset and MPII [44] to evaluate the effectiveness of the HPnet. To verify the effectiveness of our method, we first compare the proposed HPnet to the sota methods and then conduct ablation studies on the self-attention branch and the cross-branches attention module.

### 4.1. Experimental Setup

#### 4.1.1. Datasets

COCO [43] is the most typical common dataset for human pose estimation. The COCO keypoints challenge dataset consists of 118k training images and 41k testing images, and 5k valid images. The training set consists of 100k individuals annotated with 17 keypoints, which include 5 facial landmarks and 12 body joints. We train the proposed HPnet on the train set and utilize average precision on *OKS* metric to evaluate it on the validation set and test-dev set.

MPII [44] is conventional dataset for human pose estimation. Differing from the COCO dataset, the configuration of the human pose is 16 joints without facial landmarks. The dataset contains almost 15k images and 40k annotated human instances. The training set consists of 15k images and 22k individuals and the validation set contains 2729 images and 2958 persons. The *PCKh* metric is adopted to evaluate the performance of our HPnet on the MPII dataset.

#### 4.1.2. Evaluation Metrics

Object Keypoints Similarity (*OKS*) [43] is a standard metric to evaluate the keypoints distance of human instances on the COCO dataset. For each human instance, the OKS is calculated by:

(7) $$OKS=\frac{\sum_{i}\exp\left(-d_{i}^{2}/2S^{2}\sigma_{i}^{2}\right)\delta \left(v_{i}>0\right)}{\sum_{i}\delta \left(v_{i}>0\right)}.$$

Here, $d_{i}$ is the distance between the ground-truth keypoint and matched detected keypoints, and *S* is the segmentation area of this human instance. For controlling the fall-off threshold of each keypoint, the $\sigma_{i}$ is set to measure reweight of the distance.

We utilize the mean average precision (*AP*) for all instances over 10 OKS thresholds to verify the performance, and we also use the average precision over different OKS thresholds and different person scales to verify the effectiveness of the proposed method. The *AP*50 represents the percentage of keypoints in which OKS is less than 0.5, and the *AP*75 means that OKS is less than 0.75. The COCO dataset defines the scale of human instances according to the area of the bounding box of human instance; thus, we also adopt the mean average precision for all median human instances (*AP*(M)) and all large human instances (*AP*(L)) to further evaluate the performance of the proposed method.

*PCKh* [44] is percentage of correct keypoints under matching threshold as 50% of the head segment length. The *PCKh* is modified from PCK [45] and to alleviate the drawback of PCP [46] metric. For each person, the *PCKh* is calculated by:

(8) $$PCKh=\frac{\sum_{i}\delta \left(v_{i}>0\right)\delta \left(d_{i}>0.5\cdot \sigma \cdot \zeta \right)}{\sum_{i}\delta \left(v_{i}>0\right)}.$$

Here, the σ is scale bias for the MPII dataset, which is 0.6. The ζ is the diagonal length of the bounding box of the corresponding human head. We also calculate the *PCKh* of each type of keypoint to inspect the performance of our model.

#### 4.1.3. Implement Detail

The model is implemented based on the open-source toolbox MMPose [47]. For both COCO and MPII, we train the HPnet with Adam optimizer, and the learning rate is set to $1\times 10^{-4}$. We adopt a multi-step learning rate schedule to decrease the LR at {170,200}, and the total epoch to train the HPnet is set to 210. The train and test data augmentation is following the routine. For the COCO dataset, our models are trained on a host with 8 Nvidia RTX 2080 Ti GPUs, and each training process costs from 20 h for res50 with 256×192 input resolution to 90 h for res152 with 256×192 input resolution. For the MPII dataset, we train our models on 4 GPUs, and each training costs 21 h for 256×256 and 41 h for 384×384 with res152.

We conduct our model at different input image scales 256×192 and 384×288 on the COCO dataset, 256×256 and 384×384 on the MPII dataset. In the self-attention block, we set the dropout ratio as 0.1, and use the ReLU activation function. The number of heads in MHSA is set to 8. The keypoints head follows the SimpleBaseline head and adopts three deconvolutions with stride=2. The resolution of the output heatmap is 1/4 of the input image size.

Following the conventional setting [47], we adopt the same person detector [9] to generate the bounding box of each person instance across all COCO validation and test-dev sets. The person detection *AP* on the validation set is 56%, and 60.9% on the test-dev set. For the MPII dataset, we use the ground truth bounding box to evaluate all the methods.

### 4.2. Results on Coco Keypoint Detection Task

We compare our HPnet with the state-of-the-art methods on the bath valid set and test-dev set of the COCO dataset. The performance of our model is comparable to other state-of-the-art methods.

As shown in Table 1, compared to the SimpleBaseline [8] method, our HPnet obtains almost 2.4–1.3% improvement with the same convolution branch. These results indicate that the self-attention branch and fusion module in our HPnet is reasonable. Compared to the TransPose [12], our HPnet obtains the 1.1% improvement with the same convolution branch and the same number of self-attention modules. The input resolution of each self-attention module is still 1/8 of input image size in the TranPose [12]. However, the input resolution of each self-attention module is gradually decreasing from 1/8 to 1/32 in the different stages of our HPnet. This result shows that the parallel model outperforms the sequential model in this scenario. As shown in Table 2, we conduct experiments on the test-dev set to verify the effectiveness of our HPnet, and the results show that our model is comparable to other state-of-the-art models.

### 4.3. Results on MPII Dataset

We also conduct experiments on the MPII validation set to further verify the effectiveness of our HPnet. The HPnet still achieves the competitive performance of *PCKh* on the MPII dataset.

As shown in Table 3, our HPnet achieves overall 91.8% of *PCKh* on the validation set, which surpasses the TokenPose [26] and HRNet [9]. Especially on the elbows and ankles, our model achieves almost 2–3% improvement.

To investigate the high-precision localization, we evaluate our method under different matching thresholds PCKh@[0.0, 0.5] to further verify the performance of our HPnet. The PCKh@[0.0, 0.5] means that we normalized the distance of predicted keypoints and ground truth with different ratios of head size and calculate the percentage of correct keypoints.

**Figure 5:**
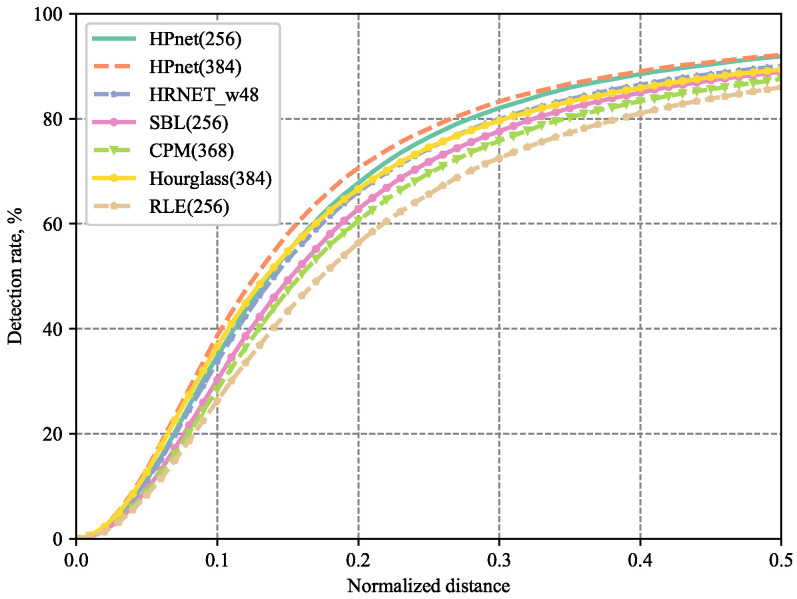
Comparisons of *PCKh*@[0.0, 0.5] on MPII validation set.

As illustrated in Figure 5, our HPnet surpasses the previous state-of-the-art methods. Especially, our HPnet achieves distinct improvement under the small matching threshold with 384 input resolution.

### 4.4. Ablation Study

#### 4.4.1. Effectiveness of the Self-Attention Branch

Compared to vanilla ResNet-based pose estimation models such as SimpleBaseline [8], the HPnet introduces the self-attention branch. In this section, we conduct ablation experiments on the configuration of the self-attention branch without any cross-branches attention modules. For convenience, we utilize the three numbers ijk to represent the number of self-attention blocks in each stage.

As shown in Table 4, the base ResNet50 model only achieves 71.6% *AP*. The *AP* slightly improved with one self-attention block in the last stage, whereas the *AP*75 fractionally declined. This shows that the conflict between the two types of dependencies corrupts the localized precision, which means the self-attention branch conduces to modeling semantic information rather than finely localization. The results also show that the resolution of the self-attention block is proportional to the *AP* improvement, and the model achieves 1% improvement while plugging one self-attention branch in the first stage.

#### 4.4.2. Effectiveness of the Cross-Branches Attention

For the initial setting, the model briefly element-wise sums the two types of features together, and the conflict corrupts the performance in the jitter. We develop the cross-branches attention module to mitigate small localized errors.

As shown in Table 5, we develop four extra-type fusion blocks for comparison. The m-* attentions are mutual attentions that generate a gated value to regulate the opposite branch, which is shown in Figure 4. The *-spatial attentions are spatial attentions which predict a $g\in R^{1\times H\times W}$ to gate the feature in all pixel locations. The concat concatenates both features and transforms the feature with a 1×1 convolution. The self-channel cross-branches attention obtains superior performance rather than others, especially in *AP*75. The proposed fusion block alleviates the degradation of high-precision localization when the self-attention blocks are plugged into the model.

Further experiments on the deeper models and large resolution of input images show that the cross-branches attention obtains impressive *AP*75 improvement. As shown in Table 6, the res101-based HPnet achieves almost 2% improvement under the *AP*75 protocol with the 384×288 input image size. Even if the res152-based HPet, the *AP*75 still increases 1%.

**Figure 6:**
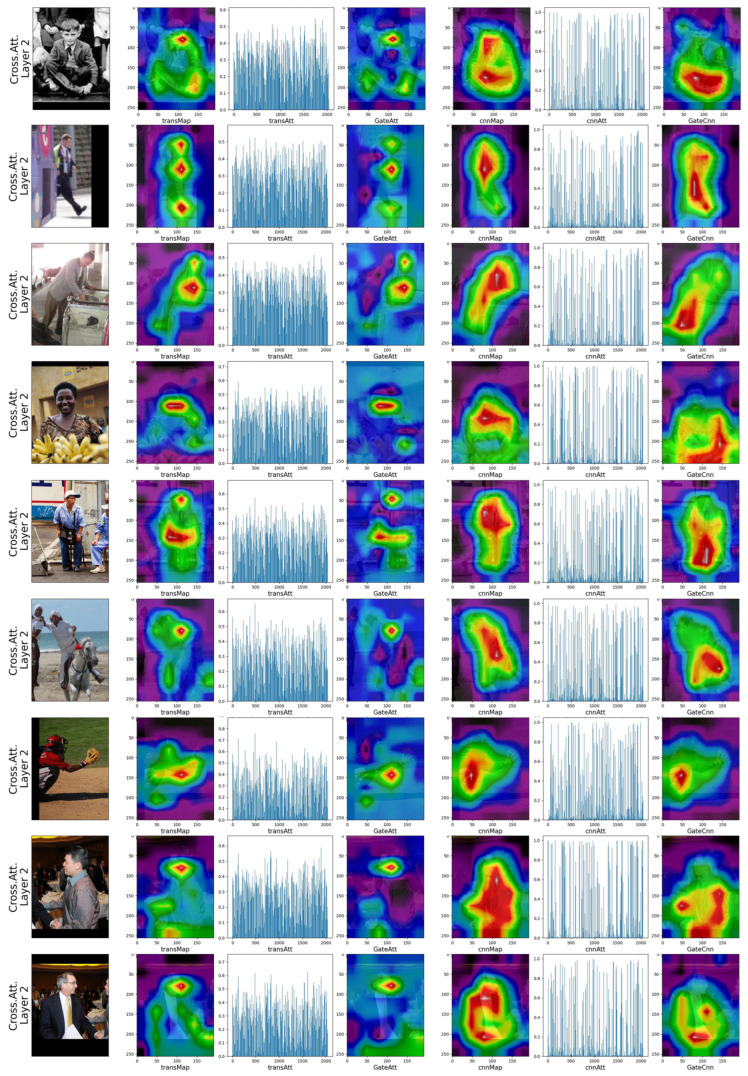
Visualization of the amplitude of the features response and the attention values at final cross-channel attention fusion block.

We also visualize the amplitude of features to further verify the effectiveness of the cross-branches block. As illustrated in Figure 6, the transmap represents the feature generated by the self-attention branch, and the transAtt is the gated value generated by the $G_{t}$. After being gated, the response map alters to complementary to the convolution branch. The convolution branch still focuses on the person instance even is followed by a gated function. Thus, the proposed HPnet drives each branch to learn complementary information rather than conflicting information.

#### 4.4.3. Hyperpramameter Tuning

Position embedding plays an important role in the transformer, and we also verify the effectiveness of position embedding. As shown in Table 7, the *AP* drops counter-intuitively if we add the position embedding in the self-attention block. The reason is that the convolution branch of HPnet models position-sensitive information, and injecting position embedding into the self-attention branch causes conflict with the other convolutional branch.

We add more self-attention blocks in the self-attention branch to conclude the saturated number of self-attention blocks for the human pose performance. However, if we add two self-attention blocks in stage 2, the Table 8 shows that more blocks slightly decrease the *AP*. The reason is that the convolutional branch is pre-trained on ImageNet, and the self-attention block is trained from scratch. Due to the gated function in the cross-attention module, the self-attention blocks are hard to train adequately.

We also conduct the dark heatmap decoding method as shown in Table 9. Our HPnet is compatible with the dark method. Our model achieves 0.5% improvement by replacing the conventional Gaussian heatmap with the dark method directly.

**Figure 7:**
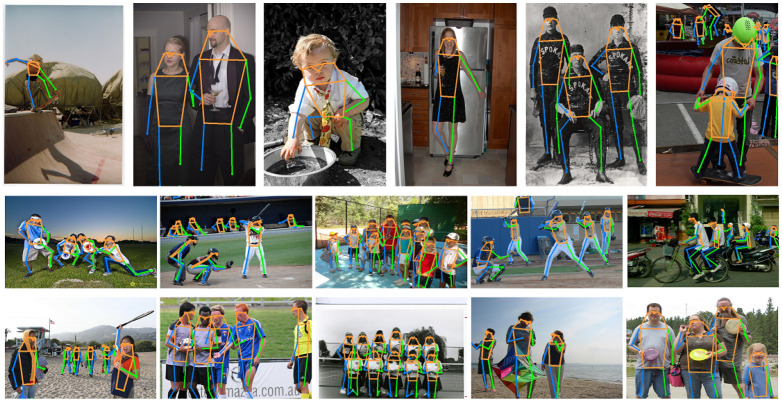
Visualization of human pose estimation results of our HPnet.

We also show the estimated poses of examples on the COCO validation set in Figure 7.

## 5. Discussion

### 5.1. Performance at Each Type of Joint

**Figure 8:**
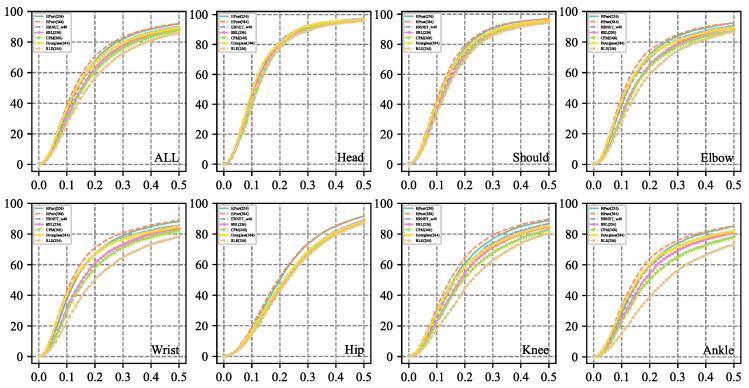
Comparison of *PCKh*@[0.0, 0.5] on each type of joints on MPII validation set.

We plot the *PCKh* at [0.0, 0.5] at each type of keypoint to inspect our model. As illustrated in Figure 8, our HPnet surpasses the previous methods on the overall *PCKh* and *PCKH* at each type of joint. Especially, our HPnet achieves considerable improvement on the challenging joints—wrist and ankle. The reason is that the hybrid parallel net applies the self-attention module to boost the semantic representation of joints that are away from the torso and possess large variations. We apply high-resolution input images to estimate human poses and attain decent improvement on high-precision localization under lower matching thresholds. We also realize that employing high-resolution features to estimate the human pose only achieves negligible improvement on *PCKh*@0.5. The reason is that the matching threshold 0.5 covers most instances except the extreme cases which are failed to detect even applying higher resolution.

### 5.2. Location Errors Analysis

We utilize the tool [50] to diagnose the location error of keypoints estimated by our HPnet on the COCO dataset. The **Good** predicted keypoints are which the *OKS* is greater than 0.85 with matched ground-truth keypoints. The overall inaccurate predicted keypoints are divided into four types: **Jitter** is that the 0.5<=oks<0.85; the **Inversion** is that the *OKS* is greater than 0.5 with mismatched keypoints; and the **Swap** means mismatched human instance. The **Miss** means *OKS* is less than 0.5 with all keypoints in this image.

**Figure 9:**
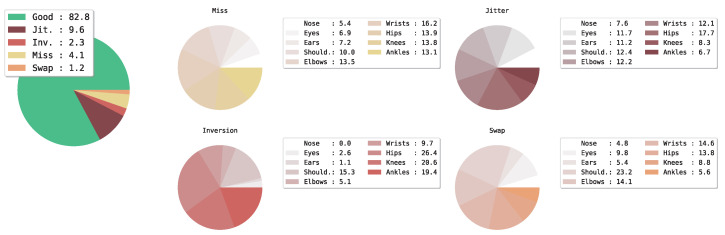
Overall performance of predicted human keypoints using our HPnet (ResNet-152) on COCO validation set.

As shown in Figure 9, even though our method mitigates the errors and achieves comparable performance with the other sota methods, our model still suffers from the Jitter error. The reasons are that the resolution of the input image is still limited by the computational complexity, and the representation of the human pose is the vanilla Gaussian heatmap. A higher-resolution heatmap or well-designed representation of the human pose may further alleviate the Jitter errors.

From the pie chart of each type of error, we observe that the distributions of Inversion and Swap error on each keypoint are inconsistent with the other two types. The Miss and Jitter are balanced to each keypoint, but the Inversion and Swap are various. The lower body of the human pose is distinguished from the upper body by Inversion, which means the hips, knees, and ankles are easier to match other keypoints. One reason is that the legs overlap each other frequently in this dataset. Differing from the Inversion, the shoulders are easier to swap to other instances, one reason is occlusion by other human instances. In addition, the precision of the bounding boxes generated by the person detector is the key role to mitigate the errors.

### 5.3. Failure Cases Analysis

We also show some failure cases in COCO validation set by our HPnet, and we summarize the cases into four types according to the diagnosis tool [50].

**Figure 10:**
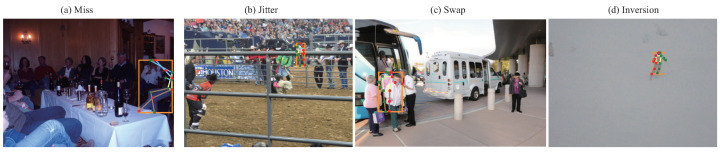
Failure cases by our HPnet (ResNet-152) on validation set.

As shown in Figure 10a, the bounding box of the human instance is partially occluded by other human instances, which may confuse the model with the corresponding part of this human instance, because the heatmap-based models generate the location of each joint by extracting the top one response of each heatmap. Although the bounding box injects visible parts of other human instances, the estimated pose of this human may be disturbed by neighbor human instances.

The Figure 10b and d show that the small and blurred instances of human instances are still the main factor to degrade the performance of human pose estimation. The small instance indicates that the details around the joints are lost; thus, accurately localizing the joints is unfeasible. In addition, the small blurred person lost the distinctness of each joint; thus, the inversion error of this scenario is inevitable.

Our HPnet still suffers from the crowding scenario as illustrated in Figure 10c. The Swap error shows that the pose of the occluded human instance is corrupted by the front human instance. The proposed method has to infer the invisible joint without any appearance information of this joint, and it is reasonable that the localization is inaccurate.

## 6. Conclusions

In this paper, we propose a Hybrid Parallel network (HPnet) to parallelize the self-attention and convolution, and a cross-branches attention block to fusion the two types of features. Our Hybrid Parallel network mitigates the mutual conflict while the HPnet leverages the complementary capabilities of convolutional modules and self-attention modules for human pose estimation. We conduct experiments on both COCO and MPII datasets to demonstrate the effectiveness of the proposed HPnet, and the extended experiments verify the effectiveness of the cross-branches attention module. In addition, the hybrid parallel model is suitable for high-precision localization vision tasks on account of the complementary capability inherited by the self-attention module and the convolution module. In the future, we will further investigate the main issues to degrade the performance of the human pose estimation model, e.g., the small person, occlusion etc., and leverage the large model and structure information of human pose to achieve higher-precision keypoints localization.

## Figures and Tables

**Table 1:** Comparisons with state-of-the-art methods on COCO validation set.

Method	Res	Backbone	*AP*	*AP*50	*AP*75	*AP*(M)	*AP*(L)
SBL [8]	256 × 192	Res50	70.4	88.6	78.3	67.1	77.2
SBL [8]	384 × 288	Res50	72.2	89.3	78.9	68.1	79.7
SBL [8]	256 × 192	Res101	71.4	89.3	79.3	68.1	78.1
SBL [8]	384 × 288	Res101	73.6	89.6	80.3	69.9	81.1
SBL [8]	256 × 192	Res152	72.0	89.3	79.8	68.7	78.9
SBL [8]	384 × 288	Res152	74.3	89.6	81.1	70.5	81.6
TransPose-R-A3 [12]	256 × 192	ResNet-S	71.7	88.9	78.8	68.0	78.6
TransPose-R-A4 [12]	256 × 192	ResNet-S	72.6	89.1	79.9	68.8	79.8
TransPose-H-A3 [12]	256 × 192	HRNet-S-W32	74.2	89.6	80.8	70.6	81.0
TransPose-H-A4 [12]	256 × 192	HRNet-S-W48	75.3	90.0	81.8	71.7	82.1
HPnet	256 × 192	Res50	72.8	90.0	80.9	65.7	75.2
HPnet	384 × 288	Res50	74.8	90.4	82.0	67.7	77.9
HPnet	256 × 192	Res101	73.3	90.4	81.4	66.3	75.7
HPnet	384 × 288	Res101	75.1	90.4	82.0	67.9	78.0
HPnet	256 × 192	Res152	73.7	90.4	81.7	66.6	76.3
HPnet	384 × 288	Res152	75.6	90.5	82.7	68.4	78.6

**Table 2:** Comparisons with state-of-the-art methods on COCO test-dev set.

Method	Res	*AP*	*AP*50	*AP*75	*AP*(M)	*AP*(L)
G-RMI [2]	353 × 257	64.9	85.5	71.3	62.3	70
Integral [48]	256 × 256	67.8	88.2	74.8	63.9	74
CPN [7]	384 × 288	72.1	91.4	80	68.7	77.2
RMPE [49]	320 × 256	72.3	89.2	79.1	68	78.6
HRNet-W32 [9]	384 × 288	74.9	92.5	82.8	71.3	80.9
HRNet-W48 [9]	384 × 288	75.5	92.5	83.3	71.9	81.5
TokenPose-L/D24 [26]	256 × 192	75.1	92.1	82.5	71.7	81.1
TokenPose-L/D24 [26]	384 × 288	75.9	92.3	83.4	72.2	82.1
SBL [8]	384 × 288	73.7	91.9	81.1	70.3	80
TransPose-H-A6 [12]	256 × 192	75.0	92.2	82.3	71.3	81.1
HPnet	384 × 288	75.4	92.6	83.2	71.8	81.2

**Table 3:** Comparisons of *PCKh* on MPII validation set.

Method	Res	Head	Shoulders	Elbows	Wrists	Hips	Knees	Ankles	*PCKh*
Hourglass [16]	256 × 256	96.6	95.6	89.5	84.7	88.5	85.3	81.9	89.4
CPM [1]	368 × 368	96.1	94.8	87.5	82.2	87.6	82.8	78.0	87.6
SBL [8]	256 × 256	96.9	95.4	89.4	84.0	88.0	84.6	81.1	89.0
HRNet-W48 [9]	256 × 256	97.2	95.7	90.6	85.6	89.1	86.9	82.3	90.1
RLE [17]	256 × 256	95.8	94.6	86.9	78.3	87.5	80.4	73.5	86.0
TokenPose-L/D24 [26]	256 × 256	97.1	95.9	90.4	86	89.3	87.1	82.5	90.2
HPnet	256 × 256	97.0	96.7	92.2	88.0	91.5	88.7	85.3	91.8

**Table 4:** Ablation study on the configuration of the self-attention branch. The convolution branch is ResNet50, and the fusion module is add.

ijk	*AP*	*AP*50	*AP*75	*AP*(M)	*AP*(L)
-	71.6	89.7	79.8	64.6	74.2
001	71.9	89.9	79.7	64.9	74.6
010	72.2	90.0	80.1	65.0	74.8
100	72.5	90.1	80.6	65.4	75.0
111	72.5	90.0	80.2	65.4	75.1

**Table 5:** Ablation study on the different feature fusion modules. The convolution branch is ResNet50, and the configuration of the self-attention branch is 111.

Method	*AP*	*AP*50	*AP*75	*AP*(M)	*AP*(L)
concat	72.3	89.8	79.9	65.3	74.8
m-spatial	72.5	90.0	80.0	65.5	74.9
m-channel	72.6	89.9	80.1	65.5	75.1
self-spatial	72.4	89.8	80.2	65.2	75.2
**CBA**	72.8	90.0	80.9	65.7	75.2

**Table 6:** Effects of the cross-branches attention on different backbones. The configuration of the self-attention branch is 111.

Backbone	CBA	*AP*	*AP*50	*AP*75	*AP*(M)	*AP*(L)
res101		73.2	89.8	80.0	65.8	76.2
-	*√*	75.1	90.4	82.0	67.9	78.0
res152		74.6	90.1	81.7	67.4	77.6
-	*√*	75.5	90.5	82.7	68.4	78.6

**Table 7:** Effect of the position embedding.

Res	*AP*	*AP*50	*AP*75	*AP*(M)	*AP*(L)
-	72.54	89.97	80.24	65.40	75.07
w/pos	72.17	89.85	79.81	65.11	74.65

**Table 8:** Effect of more self-attention blocks.

Config	*AP*	*AP*50	*AP*75	*AP*(M)	*AP*(L)
111	72.54	89.97	80.24	65.40	75.07
121	72.46	89.94	80.12	65.32	74.92

**Table 9:** With dark decoding method.

Post	*AP*	*AP*50	*AP*75	*AP*(M)	*AP*(L)
-	75.6	90.5	82.7	68.4	78.6
Dark	76.25	90.93	83.20	69.19	79.32

## Data Availability

The data presented in this study are available on request from the corresponding author.
